# Strain-Rate-Dependent Thermo-Microstructural Evolution in Fe-Mn-Si Shape Memory Alloys Under Cyclic Tensile Training Process

**DOI:** 10.3390/ma19051025

**Published:** 2026-03-06

**Authors:** Qian Sun, Bo Cao, Takeshi Iwamoto

**Affiliations:** 1 School of Civil Aviation, Northwestern Polytechnical University, Xi’an 710072, China; sunqian@nwpu.edu.cn; 2 Yangtze River Delta Research Institute of NPU, Taicang, No. 27 Zigang Road, Science and Education New Town, Taicang 215400, China; 3 Academy of Science and Technology, Hiroshima University, 1-4-1 Kagamiyama, Higashi-Hiroshima 739-8527, Japan

**Keywords:** shape recovery behavior, Fe-Mn-Si shape memory alloy, loading strain rate, martensitic transformation, thermal analysis, microstructure

## Abstract

Iron-based shape memory alloys (Fe-SMAs) are promising for structural retrofitting because of their low cost, corrosion resistance, and manufacturability. However, the effect of strain rate on the coupled thermo-microstructural evolution during cyclic training remains underexplored. In this study, samples underwent cyclic tensile training at quasi-static and impact strain rates. After each cycle, DSC was adopted to obtain transformation temperatures and enthalpies, and selected cycles were characterized by EBSD (KAM and IPF) to quantify phase fractions and variant statistics. Results show tensile loading shifts transformation temperatures, with the principal difference between regimes appearing in the evolution of martensite finish temperature. Under impact loading, the transformation enthalpy increases more rapidly (0.18 to 0.8 J/g in absolute value), and the driving force decreases more markedly by the fourth cycle (−0.0578 to −0.1117 J/g), indicating faster thermodynamic changes at high strain rates. Internal stress and dislocation storage accumulate faster under impact, lowering the effective stress (−17.01 MPa) for transformation and promoting martensite nucleation/growth. EBSD reveals increasing lattice distortion; in impact-trained samples, single-variant martensite and higher stored energy reduce interface resistance and enable elastic energy release, accelerating transformation and improving shape recovery.

## 1. Introduction

Iron-based shape memory alloys (Fe-SMAs) [1,2,3,4,5] have been extensively employed in applications such as civil infrastructure [6,7,8,9] and intelligent actuation systems [10,11] owing to their excellent shape memory effect (SME) [12,13,14], superior corrosion resistance [15], low cost [16,17], and outstanding workability [18]. Consequently, Fe-SMAs are frequently subjected to complex stress states [19] and various extreme environments [20,21,22] from low to high temperatures. However, fundamental mechanical testing of these alloys has revealed pronounced tension–compression asymmetry [22,23,24] in parameters such as stress–strain, recovery strain, transformation latent heat, and plasticity. To elucidate the underlying mechanisms of this phenomenon, researchers have applied microstructural characterization techniques including X-ray diffraction (XRD) [25,26], electron backscatter diffraction (EBSD) [26,27], and transmission electron microscopy (TEM) [28]. In situ observation methods [29,30] allow capture of transformation dynamics in real time, overcoming the limits of static imaging, but they are generally unsuitable for high strain rate loading. As an alternative, electrical resistivity measurements [31,32] have been developed to detect phase transformations in Fe-SMAs and to quantify the critical stress threshold for transformation under tensile loading. Despite their utility, these approaches mostly record observable responses from mechanically driven tests and remain more descriptive of phenomena than explanatory of the fundamental mechanisms.

In recent years, extensive investigations have been carried out on the phase transformation behavior and thermal characteristics of Fe-SMAs. Transformation kinetics addresses the question of when and how a phase transformation initiates from a mechanistic and energy-based perspective. Rasheed et al. [33] performed differential scanning calorimetry (DSC) on Fe-15Mn-10Cr-8Ni-4Si by only brine quenching and observed a pronounced decrease in $A_{s}$ (Austenite start temperature) from 366 K to 312.02 K, demonstrating that quenching effectively improves functional performance. Marinelli et al. [34] suggested that the enthalpy contribution to the total crystal energy is negligible in Fe-Mn and Fe-Mn-Co alloys. Using Gibbs free energy modeling, Baruj et al. [35] quantified the energy barrier for transformation initiation in Fe-Mn and Fe-Mn-Co alloys from 1273 to 250 K, reporting that it facilitates martensitic transformation in early cycles but suppresses it as cycling proceeds (10 cycles in total). Balagurov et al. [36] demonstrated that thermal cycling in Fe-Mn-Si alloys (which underwent cold rolling, hot rolling at 673 K, and annealing at 1273 K for 1 h, with thickness reductions ranging from 10% to 30% depending on composition) induces defect accumulation and internal stresses, resulting in a lower forward transformation temperature and a higher reverse transformation temperature, thereby enlarging the hysteresis. Similarly, Mertinger et al. [37] reported that in Fe-Mn-Cr SMAs, transformation temperatures and latent heat tend to stabilize after initial fluctuations during cycling, while increased Cr content reduces transformation temperature, hysteresis, and latent heat. Furthermore, Rao and Srinivasa [38] decomposed the response of SMA elements subjected to tensile and torsional loading into thermoelastic and dissipative contributions. By introducing two distinct Gibbs free energy potentials, they formulated a quantitative link between the thermodynamic driving force and the corresponding transformation fraction. This theoretical framework provides a basis for evaluating the stability of cyclic superelastic behavior, while accounting for thermomechanical coupling and the progressive functional degradation of SMAs. Collazo et al. [39] further demonstrated via DSC that the martensitic transformation in Fe-SMAs is not fully reversible, with reversibility dependent on strain magnitude and number of thermal cycles. By studying the kinetics of the ε-martensite→γ-austenite reverse transformation, they constructed isoconversion curves that describe transformation progress over time, thereby providing guidance for optimizing thermally activated cycling.

In the previous study, a systematic investigation of thermomechanical training of Fe-SMAs under tensile [40] loading at varying strain rates have been undertaken. The results indicate that, under identical training protocols, the shape memory response is significantly influenced by strain rate and cycle number [40]. EBSD results revealed the evolution process of a single martensitic variant during impact training. It is noteworthy that the transformation temperatures of Fe-SMAs shift upon deformation or compositional modification akin to Ni–Ti SMAs [41,42], and others mentioned above. From this perspective, the reason for the increase in the shape recovery ratio of Fe-SMA during impact tensile training process, along with the possible changes in the phase transformation temperature and driving force, remains unclear. Particularly, a comprehensive correlation among macroscopic properties (e.g., shape recovery rate), microstructural features (e.g., dislocations or twins), and transformation kinetics (e.g., internal stress, effective stress, enthalpy change, and driving force) under different loading conditions remains lacking for Fe-SMAs; the mechanistic basis of their transformation kinetics during training is still unclear.

In the present study, which builds on our prior work concerning the SME of Fe-SMAs under cyclic training process [40] in tensile loading at both quasi-static and impact strain rates, an in-depth thermodynamic analysis and microstructural analysis were conducted. First, specimens subjected to cyclic training under both quasi-static and impact tensile loading conditions were prepared after different numbers of cycles. These samples were subsequently sectioned into samples suitable for DSC measurements and EBSD characterization, followed by the corresponding tests. By systematically analyzing the evolution of transformation temperatures, the variations in transformation enthalpy and thermodynamic driving force were evaluated. In addition, the internal stress and effective stress at each cycle were determined and compared. Finally, a comprehensive interpretation was conducted in conjunction with the microstructural observations.

## 2. Methodology

### 2.1. Material and Experimental Procedure

**Figure 1:**
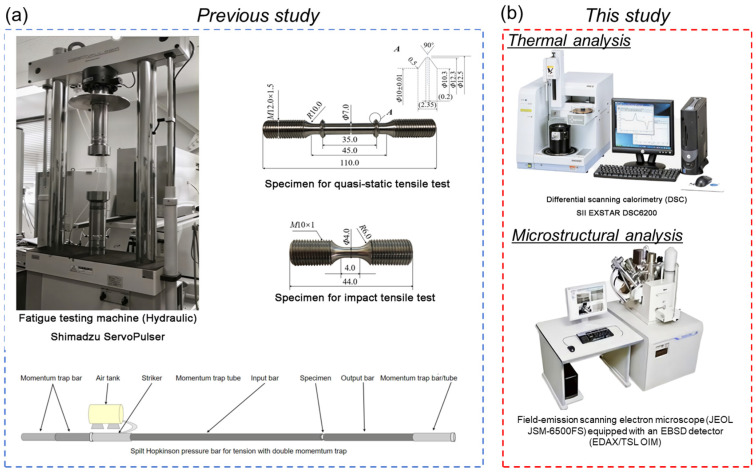
The research framework of this study. The (**a**) blue area represents the previous study [32,40], while the (**b**) red area (this study) constitutes a further analysis building upon the prior work.

In this study, a Fe-28Mn-6Si-5Cr SMA purchased from Awaji Materia Co., Ltd., Hyogo, Japan, was employed. Prior to mechanical testing, all samples underwent a solution heat treatment at 1223 K for 30 min. Figure 1 presents the research framework of this study. The sections highlighted in red represent further analyses (thermal and microstructural) building upon the previous works [32,40]. A brief overview of the previous research steps is provided here; for the training process of the Fe-SMA, six cycles of tensile loading were performed under quasi-static (0.0001 s^−1^) and impact strain rates (310 s^−1^). Each cycle included loading to 7% pre-strain, unloading at a constant rate, and thermal recovery at 873 K.

In this work, the samples after each training cycle were cut using electrical discharge machining for subsequent DSC analysis. DSC measurements were conducted using an SII EXSTAR DSC6200 system (Hitachi High-Tech Co., Ltd., Tokyo, Japan) under a nitrogen atmosphere, with both heating and cooling rates controlled at 1 K/min to obtain clearer and more distinguishable heat flow curves. The temperature range for the measurements was from 313 K to 473 K during heating, and subsequently cooled down to 193 K. The transformation temperatures were determined using Muse Measurement v 9.21U software. The determination of enthalpies was carried out using a T-Slice Analysis (Integral Tangential) taking into account the baseline shift and nonlinearity. In addition, EBSD analysis was performed to investigate the underlying mechanisms. EBSD measurements were conducted using a field-emission scanning electron microscope (JEOL JSM-6500FS, from Jeol Ltd., Tokyo, Japan) equipped with an EBSD detector (EDAX/TSL OIM, from EDAX, Inc., Tokyo, Japan). The EBSD measurements were conducted at an acceleration voltage of 25 kV. It should be noted that 13 samples were used for analysis: 1 original sample without training, 6 samples under quasi-static training and 6 under impact training. The samples after each training cycle were analyzed by DSC and the samples after cycles 2, 4 and 6 by EBSD analysis.

### 2.2. Chemical and Mechanical Procedure

Before the DSC tests, the sample were cleaned with an appropriate amount of alcohol to remove surface oxides and other impurities. Prior to the EBSD measurements, the sample surfaces were mechanically and electrolytically polished. The electrolytic polishing was performed in a solution consisting of 90% acetic acid and 10% perchloric acid at a current of 2 A for 120 s. The EBSD measurements were conducted at an acceleration voltage of 25 kV.

## 3. Results and Discussions

### 3.1. DSC Analysis

**Figure 2:**
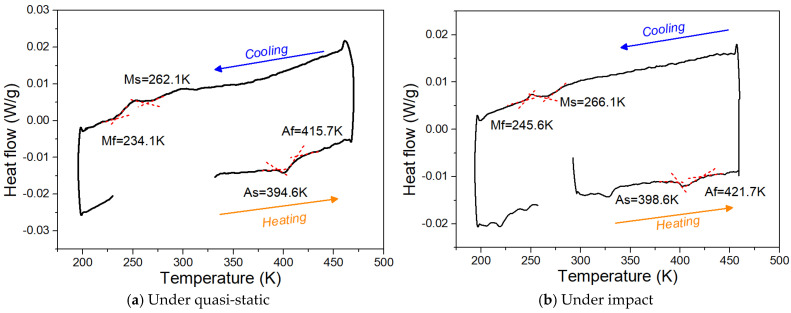
Examples of variation in heat flow with temperature during DSC analysis of the sample after the sixth training cycle under the (**a**) quasi-static and (**b**) impact tensile training process.

As mentioned above, DSC tests were performed on the specimens after each cycle; therefore, a total of thirteen datasets were obtained. Essentially, apart from the phase transformation temperatures, no noticeable differences were observed in the evolution trend of the heat flow. Therefore, to present the variation in heat flow and to illustrate the method used to determine the phase transformation temperature, Figure 2 shows two examples of variation in heat flow with temperature during DSC analysis of the sample after the sixth training cycle under the quasi-static and impact tensile training process. Two peaks corresponding to the heat exchange during forward and reverse martensitic transformation can be observed. In the figure, the determined $A_{s}$, $A_{f}$ (Austenite finish temperature), $M_{s}$ (Martensite start temperature), and $M_{f}$ (Martensite finish temperature) by the conventional intersection between two tangential lines drawn with red dashes are also shown. The unobvious $M_{f}$ in Fe-SMA have been previously reported by Tasaki et al. [43]. In this case, the second derivative can be used to further verify the specific values.

**Figure A3:**
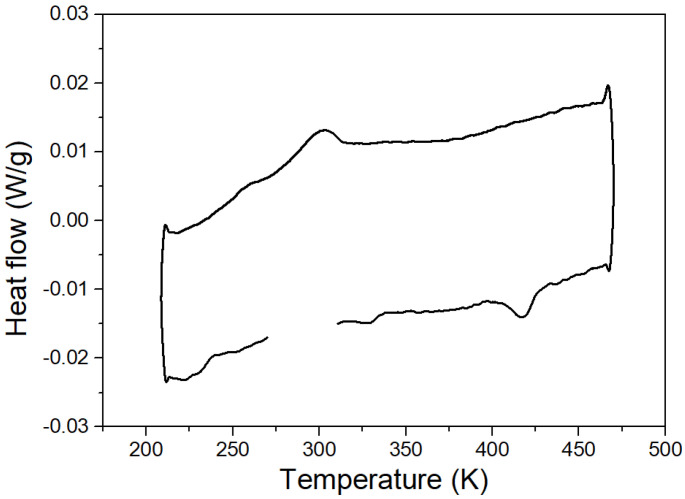
The variation in heat flow with temperature of original sample.

In general, DSC measurements should include two heating–cooling cycles, in which the first cycle erases the thermal history of the material and the second reflects its intrinsic thermal behavior. However, for the trained Fe-SMA investigated in this study, which underwent a complete training procedure including loading, unloading, and subsequent heating, our objective was to record the as-trained thermal response, i.e., the state immediately after the cyclic training process, and to clarify how the training process influences the evolution of the phase transformation temperatures of Fe-SMA. Moreover, prior to finalizing the DSC testing protocol, a preliminary test was performed on the Fe-SMA after the first training cycle. This pre-test consisted of two consecutive heating–cooling cycles. As shown in Appendix A Figure A3, no noticeable differences were observed in the transformation temperatures or transformation enthalpies between the two cycles. Therefore, a single heating–cooling cycle was adopted to identify the evolution of the transformation temperatures. In addition, it should be noted that the specimens used in this study had already been validated in our previous work and demonstrated excellent reproducibility. Therefore, the DSC measurement after each training cycle in the present study was conducted on a single specimen. Furthermore, considering that the purpose was to record the as-trained thermal response, no statistical analysis was performed. Furthermore, no additional correction was applied to the DSC results.

**Figure 3:**
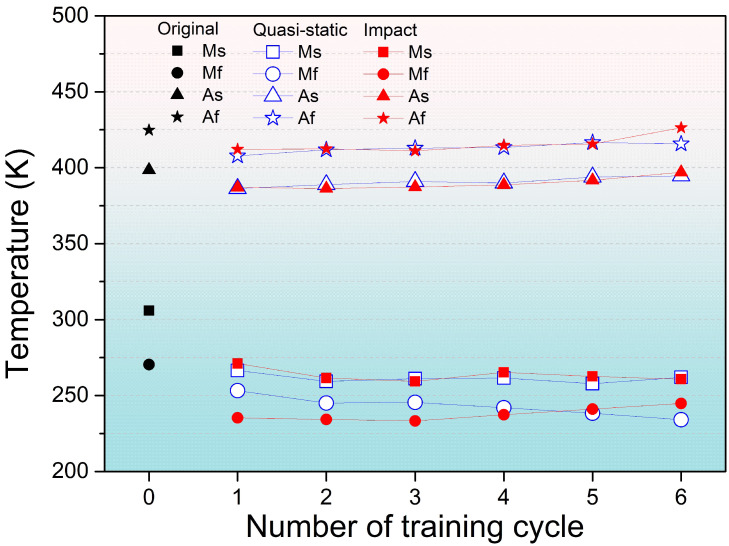
The transformation temperatures after each training cycle with respect to the number of training cycles.

Subsequently, like the above, DSC analyses are performed on the samples after each training cycle. The results on the calorimetric signatures of phase transformation with respect to the number of training cycles under the quasi-static and impact tensile training processes are illustrated in Figure 3. The values of $A_{s}$, $A_{f}$, $M_{s}$, and $M_{f}$ of samples were also summarized in Table A2. In the tensile training process, it is evident that all measured phase transformation temperatures following deformation are reduced relative to those of the undeformed reference sample, with $M_{s}$ and $M_{f}$ temperatures exhibiting the most pronounced decreases. This shift likely arises from the introduction of residual stresses and/or the increase in dislocations, both of which tend to depress transformation temperatures. As the training proceeds, the evolution of $M_{s}$, $A_{s}$, and $A_{f}$ under quasi-static and impact loading follows a similar trajectory; by contrast, the evolution of $M_{f}$ diverges most markedly between the two loading regimes. Similarly to the previous study [44] on the same Fe-SMA, both tensile loading in the present work and compressive loading in the previous work [44] exhibit comparable variations in phase transformation temperatures; both of them show the only distinction observed in the $M_{f}$ temperature. This consistency further confirms the validity and reliability of the experimental approach adopted in this study.

Specifically, $M_{f}$ decreases progressively under quasi-static tension—likely the result of increased dislocation density from repeated deformation suppressing the martensitic transformation—whereas, under impact tension, $M_{f}$ is lower than the quasi-static value after the first cycle but then rises with continued training, exceeding the quasi-static $M_{f}$ in the fifth and sixth cycles. As revealed in Ref. [40], this increase may be linked to impact-induced variant selection. Such anomalous $M_{f}$ responses reflect the direct coupling between internal defects and external loading conditions, and warrant further investigation through detailed microstructural characterization.

**Figure 4:**
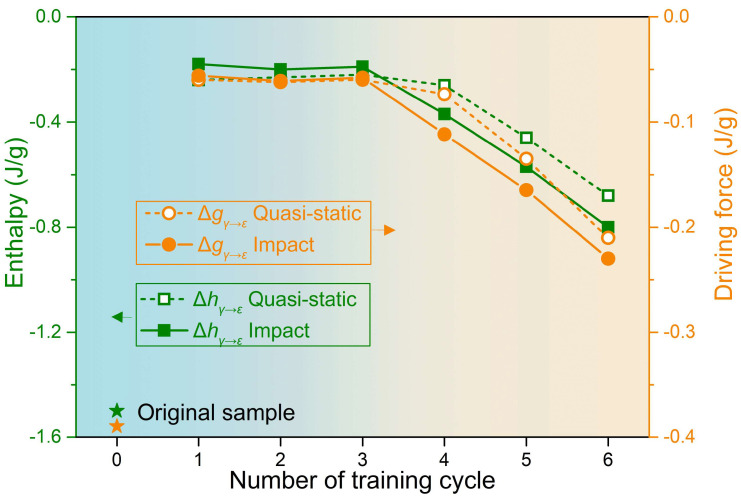
The reaction enthalpy ${∆h}_{\gamma \to \varepsilon }$ of forward martensitic transformation and driving force ${∆g}_{\gamma \to \varepsilon }$ with respect to number of training cycles under tensile training processes at the quasi-static and impact loading strain rate. The values of original samples are also indicated as stars.

The definitions of the reaction-specific enthalpy ${∆h}_{\gamma \to \varepsilon }$ and driving force ${∆g}_{\gamma \to \varepsilon }$ of the forward martensitic transformation can be found in Ref. [44]. Briefly, ${∆h}_{\gamma \to \varepsilon }$ corresponds to the area between the reaction peak and the interpolated baseline. ${∆g}_{\gamma \to \varepsilon }$ is the difference between the specific Gibbs free energy at the $M_{f}$ temperature. Figure 4 shows the ${∆h}_{\gamma \to \varepsilon }$ and ${∆g}_{\gamma \to \varepsilon }$ values under tensile training, plotted as functions of training cycle. The ${∆h}_{\gamma \to \varepsilon }$ and ${∆g}_{\gamma \to \varepsilon }$ of original sample are calculated as −1.5 and −0.390 J/g, respectively. The absolute values of ${∆h}_{\gamma \to \varepsilon }$ and ${∆g}_{\gamma \to \varepsilon }$ are discussed hereafter. Compared to the original sample, the lower ${∆h}_{\gamma \to \varepsilon }$ and ${∆g}_{\gamma \to \varepsilon }$ obtained after training may be attributed to the combined effects of microstructural defect accumulation induced by plastic deformation, residual stress, and narrowing of the transformation temperature interval [44]. During the first three cycles, ${∆h}_{\gamma \to \varepsilon }$—whether under quasi-static or impact tensile loading—remains relatively low and stable, with the corresponding ${∆g}_{\gamma \to \varepsilon }$ also almost constant. At this stage, $\varepsilon_{r}$ (recovery strain during heating process) under quasi-static tensile training already increases rapidly to 0.0416 as shown in Table A1, and maximum $\varepsilon_{r}$ under impact is only 0.0283 also as shown in Table A1, suggesting a slightly higher ${∆h}_{\gamma \to \varepsilon }$ and ${∆g}_{\gamma \to \varepsilon }$ contribute to enhancing the shape recovery behavior. By the fourth cycle, a notable increase in absolute value of ${∆h}_{\gamma \to \varepsilon }$ is observed under both loading conditions (from 0.22 to 0.26 J/g under quasi-static loading, and from 0.19 to 0.37 J/g under impact loading), accompanied by a clear increase in ${∆g}_{\gamma \to \varepsilon }$ (from 0.059 to 0.074 J/g under quasi-static loading, and from 0.058 to 0.112 J/g under impact loading). Accordingly, $\varepsilon_{r}$ under quasi-static tensile training slightly decreases to 0.0390, compared to the third cycle, attributed to lower ${∆h}_{\gamma \to \varepsilon }$, and the reorganization and/or disappearance of defect structure [45] which temporarily delays the expansion of the reversibly transformable martensite volume. Conversely, $\varepsilon_{r}$ under impact tensile training rises sharply from 0.0283 to 0.0426 in the fourth cycle. This surge aligns precisely with the marked increasing ${∆h}_{\gamma \to \varepsilon }$. As a result, a greater fraction of reversible ε-martensite is activated, leading to a substantial improvement in $\varepsilon_{r}$. In the fifth and sixth cycles, ${∆h}_{\gamma \to \varepsilon }$ and ${∆g}_{\gamma \to \varepsilon }$ under quasi-static training continue to increase, but $\varepsilon_{r}$ plateaus around 0.045. This indicates that beyond the fifth cycle, ${∆h}_{\gamma \to \varepsilon }$ and ${∆g}_{\gamma \to \varepsilon }$ can no longer significantly affect the shape recovery, suggesting that the volume fraction of reversible ε-martensite has reached its upper limit at the microscale. Under impact tensile training, ${∆h}_{\gamma \to \varepsilon }$ and ${∆g}_{\gamma \to \varepsilon }$ also continue to increase. In this case, martensitic transformation is greatly promoted and $\varepsilon_{r}$ exceeds that of the quasi-static case and reaches as high as 0.056. This implies that the microstructure under impact tensile training requires more cycles to reach saturation, and that sufficiently high ${∆h}_{\gamma \to \varepsilon }$ can drive an even larger volume fraction of ε-martensite to participate in the reverse transformation.

Moreover, the evolution of effective stress $\sigma_{eff}$ and internal stress $\sigma_{int}$ of Fe–SMA were evaluated. The derivation process of $\sigma_{eff}$ and $\sigma_{int}$ can be found in Ref. [44]. $\sigma_{int}$ represents the net mechanical stress at the phase boundary to overcome the thermodynamic equilibrium barrier, excluding the influence of $\sigma_{eff}$ on the transformation driving force.

**Figure 5:**
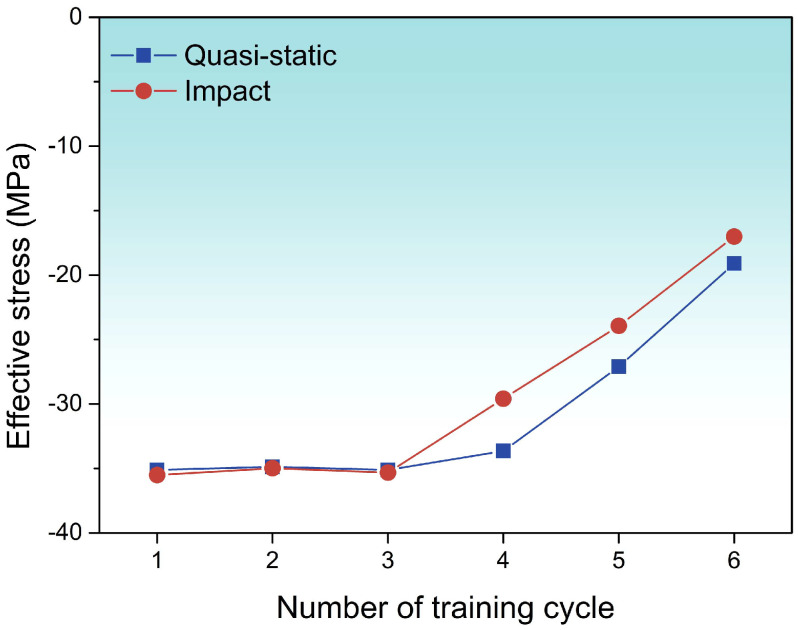
The relationship between effective stress $\sigma_{eff}$ and number of training cycle under quasi-static and impact tension.

Therefore, the relationship between $\sigma_{eff}$ and number of training cycles under quasi-static and impact tension can be obtained as shown in Figure 5. After deformation, $\sigma_{eff}$ under both conditions stays almost constant at the first three training cycles. As the number of training cycles increases, $\sigma_{eff}$ under impact is smaller than that under quasi-static loading, indicating that martensitic transformation is more easily completed during impact tension and a continuous promotion of martensitic transformation can be observed. This is consistent with $\varepsilon_{r}$ shown in Table A1, as well as the previously obtained volume resistivity results [40].

**Figure 6:**
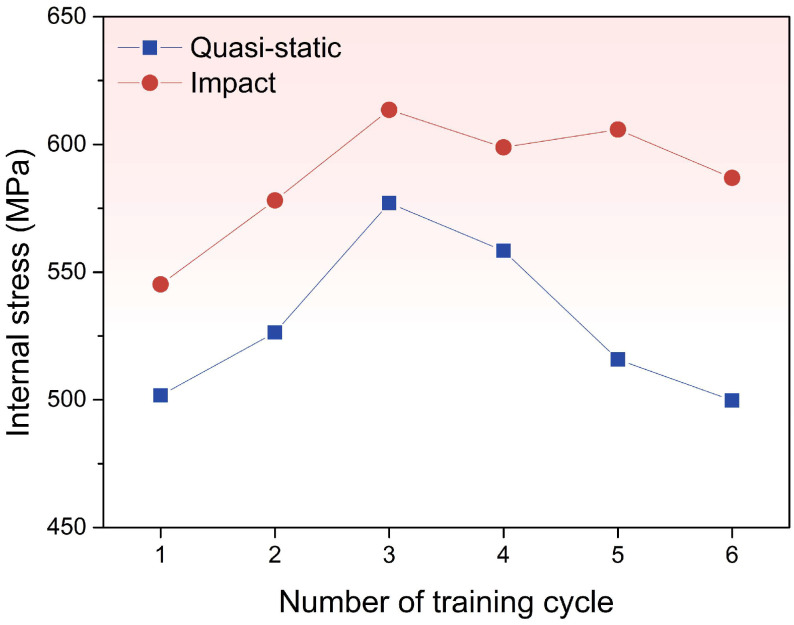
The relationship between internal stress $\sigma_{int}$ and number of training cycle under quasi-static and impact tension.

Figure 6 presents the relationship between $\sigma_{int}$ and number of training cycles under quasi-static and impact tension. Tensile $\sigma_{int}$ increases during the first three cycles, indicating that the martensitic transformation gradually becomes progressively easier to occur. In the fourth cycle and after, however, $\sigma_{int}$ varies to different levels. Specifically, $\sigma_{int}$ under the impact tension increases and stays higher, while $\sigma_{int}$ under the quasi-static tension decreases significantly. This indicates that under impact tensile loading, the martensitic transformation is continuously promoted, whereas under quasi-static loading it is suppressed. This pronounced distinction can effectively explain the difference in $\varepsilon_{r}$ (Table A1) between the two conditions.

### 3.2. Microstructural Analysis

**Figure 7:**
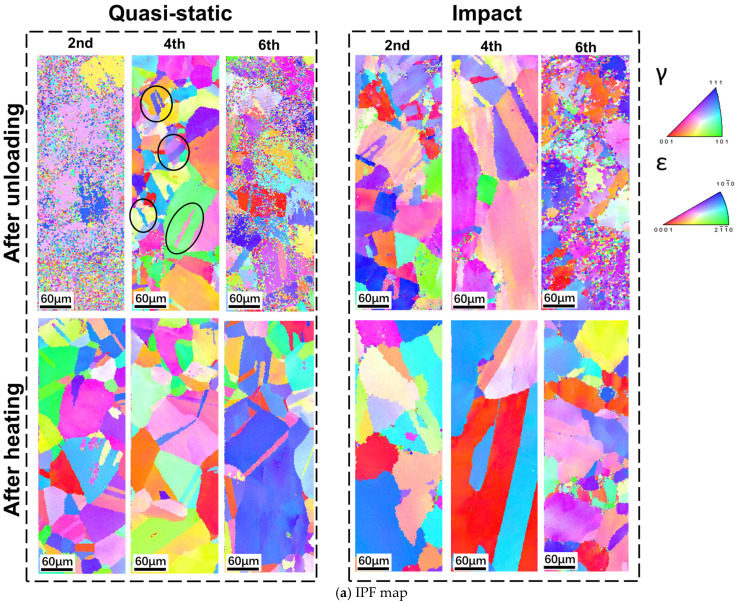

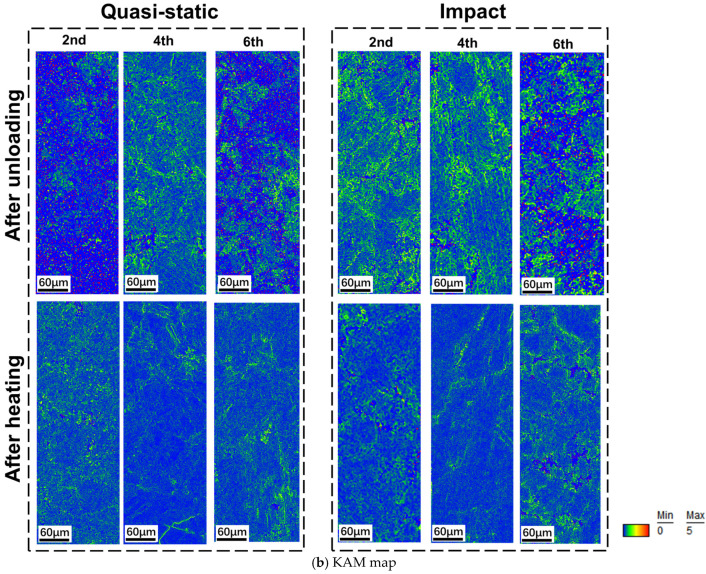
EBSD (**a**) IPF map and (**b**) KAM map of samples at the end of the second, fourth, and sixth training cycles under the quasi-static and impact tension. The specific training cycle is marked at the top.

Figure 7 presents the EBSD inverse pole figure (IPF) and Kernel Average Misorientation (KAM) maps at the end of the second, fourth, and sixth cycles. Only sparse distribution of ε-martensite appears after unloading in the second cycle. In the fourth cycle under quasi-static tension, locally concentrated ε-martensite emerges and most grains are clearly segmented as indicated in black circle; as higher $\sigma_{int}$ is observed (Figure 5), crossed multi-variant martensite induced by external quasi-static tensile load suppresses the reverse transformation. Consequently, some residual ε-martensite persists after heating, limiting the improvement on the shape recovery. Under impact tensile loading during the fourth cycle, a similar ε-martensite distribution is observed upon unloading, but lower $\sigma_{int}$ and lower ${∆g}_{\gamma \to \varepsilon }$, together with the single-variant martensite induced by external impact tensile stress, jointly promote the phase transformation, so significantly higher shape recovery (Table A1) is obtained. The formation of single and crossed multi-variants can also be found in Ref. [40] and they have been well discussed deeply. By the sixth quasi-static tensile cycle, ε-martensite is more widely and uniformly distributed with its volume fraction approaching its maximum; consequently, only a few martensitic regions remain after heating. Under impact tensile loading in the sixth cycle, the post-unloading and heated martensite distribution mirrors that of the quasi-static case. At this stage, $\sigma_{int}$ still decreases and the increased amount of single-variant martensite further promotes the reverse transformation, resulting in the maximum shape recovery (Table A1).

From the KAM maps, it is also evident that the accumulated dislocation density and local lattice distortion within the material increase progressively with each cycle. Regions exhibiting higher KAM values typically correspond to greater concentrations of irreversible dislocations, deformation twins, and retained ε-martensite, all of which collectively determine the magnitude of shape recovery. Under quasi-static tensile training, dislocations are observed to increase markedly with each cycle and then partially annihilate upon subsequent heating. However, under impact tensile loading, dislocation storage energy after unloading is higher than quasi-static loading. Combined with Figure 6, the simultaneous increase in $\sigma_{int}$ and dislocation storage energy leads to greater dislocation accumulation, more severe plasticity, and an increase in true residual strain $\varepsilon_{u}$ (which is the true strain at the end of unloading, and can also be calculated from Table A1), which suppresses shape recovery. However, the higher $\sigma_{int}$ can release part of its elastic energy during loading, thereby facilitating martensitic transformation. When combined with the externally applied tensile stress, it more readily initiates or advances the martensitic transformation. Moreover, regions of high dislocation density generate local stress concentrations that further facilitate the transformation and improve reversibility. Under external impact tensile loading, the emergence of more single-variant martensite reduces the resistance to interface migration during heating. Additionally, the stress concentrations caused by the mismatch between dislocations and sub-grain orientations act as “seeds” for transformation, making the reverse transformation easier to trigger under external tensile stress. The observed further enhancement of shape recovery corroborates this mechanism.

Briefly speaking, under the impact tensile training, the single variant is generated, so the $\sigma_{eff}$ becomes larger and $\sigma_{int}$ becomes lower, which promoted the martensitic transformation, and $\varepsilon_{r}$ becomes larger. Meanwhile, for the quasi-static tensile training, the multi-variant is generated, so lower $\sigma_{eff}$ and higher $\sigma_{int}$ suppress the martensitic transformation, ultimately lowering $\varepsilon_{r}$.

## 4. Conclusions

In this study, first, specimens subjected to cyclic training under both quasi-static and impact tensile loading conditions were prepared after different numbers of cycles based on the previous work. These samples were subsequently sectioned into samples suitable for DSC measurements and EBSD characterization, followed by the corresponding tests. By systematically analyzing the evolution of transformation temperatures, the variations in transformation enthalpy, driving force, internal stress, and effective stress were evaluated. Finally, a comprehensive interpretation was conducted in conjunction with the microstructural observations by EBSD. The principal findings are as follows:

1. The phase transformation temperatures of the samples shifted under tensile loading. The critical distinction between quasi-static and impact loading lies in the trend of $M_{f}$. Under the quasi-static loading, $M_{f}$ changes from 253.2 to 234.1 K, while under the impact loading, $M_{f}$ changes from 235.3 to 244.8 K.
2. Under impact tensile loading, the transformation enthalpy increases more steeply (0.18 to 0.8 J/g in absolute value) and the transformation driving force decreases more markedly by the fourth cycle (−0.0578 to −0.1117 J/g) than under quasi-static loading, indicating a more rapid alteration in the transformation thermodynamics at high strain rates.
3. Internal stress accumulates more rapidly during impact loading (545.21 to 613.53 MPa), thereby facilitating martensite nucleation and growth; concomitantly, the apparent effective stress required to trigger transformation is lower under impact conditions (−17.01 MPa at the lowest), further evidencing the promoting effect of high strain rates on shape recovery.
4. EBSD KAM maps show accumulating dislocations and lattice distortion, and under impact loading the higher $\sigma_{int}$, dislocation-stored energy, and single-variant martensite lower interface resistance and release elastic energy, synergistically promoting martensitic transformation and enhancing shape recovery.

Overall, the coupling of thermal driving forces, internal stress, and strain-rate-induced microstructural evolution underscores the importance of mechanically assisted transformation kinetics in Fe-SMAs. In addition, it should be noted that six cycles are still insufficient from an engineering standpoint. However, a previous study [40] confirmed that increasing the number of cycles (to nine) did not further improve the shape recovery ratio. In this case, the fatigue test may further alter the transformation temperatures, but this requires further investigation. Furthermore, tensile tests to failure should be performed before and after training (under both loading modes) to verify whether the enhanced shape recovery under impact loading is achieved without compromising ductility or operational reliability.

## Figures and Tables

**Figure A1:**
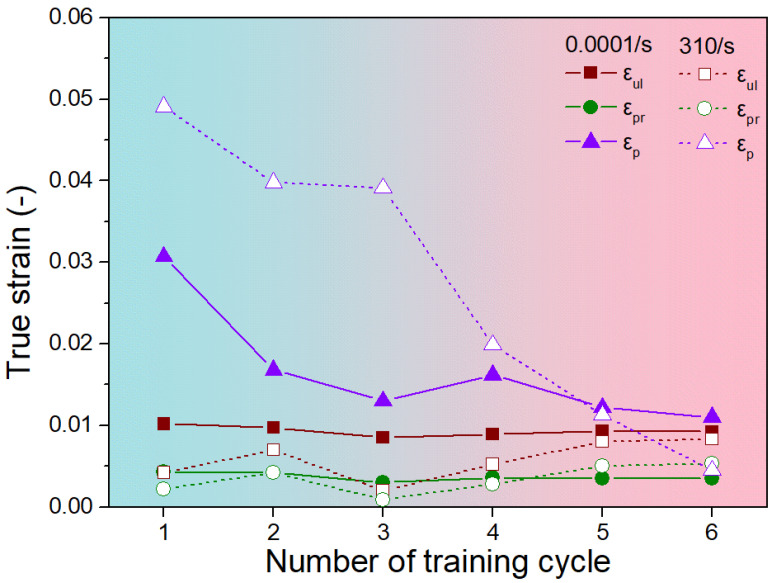
A summary of $\varepsilon_{ul}$, $\varepsilon_{pr}$, and $\varepsilon_{p}$ with respect to number of training cycles under tensile training process with a pre-strain of 7% at different loading strain rates. Data is extracted from previous studies [40] by the permission of Elsevier.

**Figure A2:**
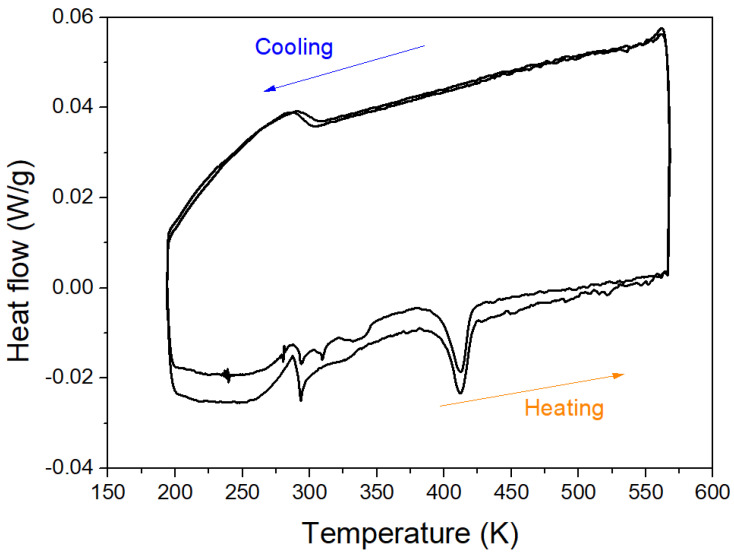
DSC curves of Fe-SMA after the deformation with two heating–cooling cycles.

**Figure A4:**
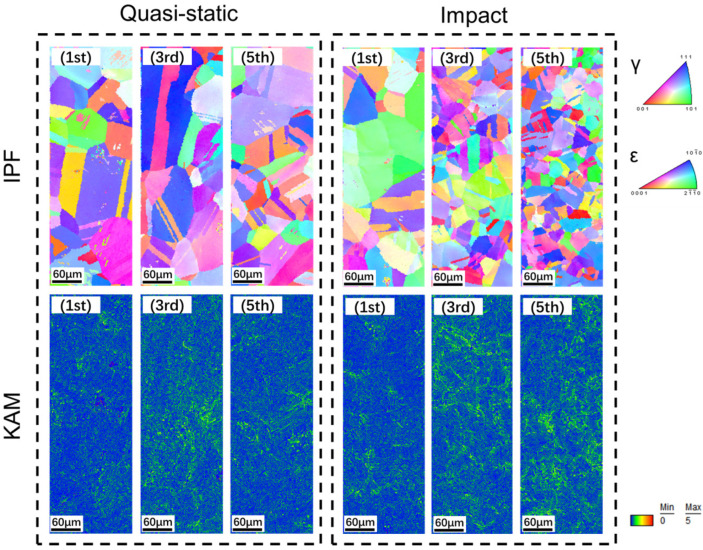
EBSD IPF and KAM maps of samples at the end of the first, third, and fifth training cycles under the quasi-static and impact tension after heating. The specific training cycle is marked at the top.

## Data Availability

The original contributions presented in this study are included in the article. Further inquiries can be directed to the corresponding authors.

## Nomenclature

Fe-SMAs	Iron-based shape memory alloys
SME	Shape memory effect
XRD	X-ray diffraction
EBSD	Electron backscatter diffraction
TEM	Transmission electron microscopy
DSC	Differential scanning calorimetry
$A_{s}$	Austenite start temperature
$A_{f}$	Austenite finish temperature
$M_{s}$	Martensite start temperature
$M_{f}$	Martensite finish temperature
$\varepsilon_{r}$	Recovery strain during heating process
${∆h}_{\gamma \to \varepsilon }$	Reaction-specific enthalpy of the forward martensitic transformation
${∆g}_{\gamma \to \varepsilon }$	Driving force of the forward martensitic transformation
$\sigma_{eff}$	Effective stress
$\sigma_{int}$	Internal stress
IPF	Inverse pole figure
KAM	Kernel average misorientation
$\varepsilon_{u}$	True strain at the end of unloading
$\sigma_{max}$	Maximum value of stress during loading
$\varepsilon_{ul}$	Recovered strain during the unloading stage
$\varepsilon_{pr}$	Pseudoelastically recovered strain during the unloading process
$\varepsilon_{p}$	Plastic strain

## Appendix A

The maximum stress $\sigma_{max}$ refers to the peak value of stress during loading. The recovered strain during the unloading stage $\varepsilon_{ul}$ is the difference between the maximum strain and $\varepsilon_{u}$. A tangent is drawn starting from the unloading stage, and the difference between the corresponding strain value on this tangent and $\varepsilon_{u}$ is defined as the pseudoelastically recovered strain $\varepsilon_{pr}$. The plastic strain $\varepsilon_{p}$ is defined as the difference between the strain at the end of heating and the initial strain. Table A1 summarizes the $\sigma_{max}$, $\varepsilon_{ul}$, $\varepsilon_{pr}$, $\varepsilon_{r}$, and $\varepsilon_{p}$ of Fe-SMA under tensile training process [40] with a pre-strain of 7% at different loading strain rates, respectively. For a more intuitive comparison, a comparison chart including $\varepsilon_{ul}$, $\varepsilon_{pr}$, and $\varepsilon_{p}$ was plotted, as shown in Figure A1. For tensile loading, $\varepsilon_{ul}$ and $\varepsilon_{pr}$ remain relatively stable under quasi-static conditions, while under impact loading, both $\varepsilon_{ul}$ and $\varepsilon_{pr}$ show initial fluctuations followed by an increasing trend. This suggests that martensitic transformation at the quasi-static loading tends toward equilibrium, whereas higher strain rate enhance reversible martensitic transformation. In addition, $\varepsilon_{p}$ under the quasi-static shows a decreasing trend and tends to stabilize by the third cycle. Under the impact loading, although $\varepsilon_{p}$ is higher than the quasi-static result during the first four cycles, it continues to decrease and eventually becomes much lower than $\varepsilon_{p}$ under the quasi-static. This indicates that cyclic impact loading can effectively eliminate some plastic defects. Previous studies [40,46] have shown that this is due to the development of crystallographic texture—where a large number of martensite variants align in a single direction, promoting martensitic transformation.

**Table A1:** A summary of $\sigma_{max}$, $\varepsilon_{ul}$, $\varepsilon_{pr}$, $\varepsilon_{r}$, and $\varepsilon_{p}$ at different loading strain rates under tensile training process with a pre-strain of 7%. Data is extracted from previous study [40] by the permission of Elsevier.

Loading Strain Rate (s^−1^)	Number of Training Cycle	$\sigma_{max}$ (MPa)	$\varepsilon_{ul}$	$\varepsilon_{pr}$	$\varepsilon_{r}$	$\varepsilon_{p}$
0.0001	1	466.6	0.0102	0.0043	0.0228	0.0307
	2	491.5	0.0097	0.0042	0.0374	0.0168
	3	541.9	0.0085	0.0030	0.0416	0.0130
	4	524.7	0.0089	0.0036	0.0390	0.0162
	5	488.7	0.0093	0.0035	0.0426	0.0122
	6	480.6	0.0092	0.0035	0.0449	0.0110
310	1	509.7	0.0042	0.0022	0.0159	0.0491
	2	543.1	0.0070	0.0042	0.0225	0.0398
	3	578.2	0.0020	0.0009	0.0283	0.0391
	4	569.3	0.0052	0.0028	0.0426	0.0199
	5	581.9	0.0080	0.0050	0.0487	0.0113
	6	569.9	0.0083	0.0053	0.0560	0.0045

Figure A2 shows the preliminary test to confirm the DSC testing protocol of deformed Fe-SMA. This pre-test included two consecutive heating–cooling cycles. No noticeable differences were observed in the transformation temperatures or transformation enthalpies between the two cycles. Therefore, a single heating–cooling cycle was adopted to record the evolution of the transformation temperatures.

**Table A2:** A summary of $A_{s}$, $A_{f}$, $M_{s}$, $M_{f}$, and ${∆h}_{\gamma \to \varepsilon }$ of samples with each condition.

Loading Strain Rate (s^−1^)	Number of Training Cycles	$M_{s}$ (K)	$M_{f}$ (K)	$A_{s}$ (K)	$A_{f}$ (K)	${∆h}_{\gamma \to \varepsilon }$ (J/g)
/	Original Sample	305.9	270.4	398.5	424.7	−1.5
0.0001	1	266.5	253.2	386.3	407.8	−0.24
	2	259.3	245.1	388.9	411.8	−0.23
	3	261.1	245.5	390.9	412.9	−0.22
	4	261.5	242	389.9	413.3	−0.26
	5	257.8	238.3	393.8	416.6	−0.46
	6	262	234.1	394.6	415.7	−0.68
310	1	271.1	235.3	387	412.1	−0.18
	2	261.5	234.3	386.3	412.4	−0.2
	3	259.3	233.3	387.3	411.1	−0.19
	4	265.3	237.4	388.7	414.7	−0.37
	5	262.6	241.1	391.7	415.6	−0.57
	6	260.7	244.8	397	426.3	−0.8

**Table A3:** A summary of ${∆g}_{\gamma \to \varepsilon }$, $\sigma_{eff}$, and $\sigma_{int}$ of samples with each condition.

Loading Strain Rate (s^−1^)	Number of Training Cycles	${∆g}_{\gamma \to \varepsilon }$ (J/g)	$\sigma_{eff}$ (MPa)	$\sigma_{int}$ (MPa)
/	Original Sample	−0.3897	/	/
0.0001	1	−0.0598	−35.11	501.71
	2	−0.0620	−34.87	526.37
	3	−0.0597	−35.12	577.02
	4	−0.0735	−33.65	558.35
	5	−0.1349	−27.11	515.81
	6	−0.2102	−19.10	499.70
310	1	−0.0560	−35.51	545.21
	2	−0.0609	−34.99	578.09
	3	−0.0578	−35.33	613.53
	4	−0.1117	−29.59	598.89
	5	−0.1647	−23.94	605.84
	6	−0.2299	−17.01	586.91

Figure A3 shows the variation in heat flow with temperature of original sample.

Figure A4 shows EBSD IPF and KAM maps of samples at the end of the first, third, and fifth training cycles under the quasi-static and impact tension after heating. In conjunction with Figure 7, it can be observed that no significant difference is evident after the first training treatment, whereas a pronounced change emerges after the second cycle, characterized by the formation of single variants. This evolution persists from the second to the sixth cycle.
